# Appraisal of Comparative Therapeutic Potential of Undoped and Nitrogen-Doped Titanium Dioxide Nanoparticles

**DOI:** 10.3390/molecules24213916

**Published:** 2019-10-30

**Authors:** Muhammad Arslan Ahmad, Yang Yuesuo, Qiang Ao, Muhammad Adeel, Zhang Yan Hui, Rabia Javed

**Affiliations:** 1 Department of Tissue Engineering, China Medical University, Shenyang 110122, China; arslan.slu@gmail.com (M.A.A.); zhangyanhui@cmu.edu.cn (Z.Y.H.); 2 Key Lab of Eco-restoration of Regional Contaminated Environment, Shenyang University, Ministry of Education, Shenyang 11044, China; 3 Beijing Key Laboratory of Farmland Soil Pollution Prevention and Remediation, College of Resources and Environmental Sciences, China Agricultural University, Beijing 100193, China; chadeel969@gmail.com

**Keywords:** biomedicine, TiO_2_ nanoparticles, X-ray diffraction, transmission electron microscopy, N-doping, antibacterial and antifungal activity, alpha amylase inhibition, protein kinase inhibition

## Abstract

Nitrogen-doped and undoped titanium dioxide nanoparticles were successfully fabricated by simple chemical method and characterized using x-ray diffraction (XRD), scanning electron microscopy (SEM), energy dispersive x-ray (EDX), and transmission electron microscopy (TEM) techniques. The reduction in crystalline size of TiO_2_ nanoparticles (from 20–25 nm to 10–15 nm) was observed by TEM after doping with N. Antibacterial, antifungal, antioxidant, antidiabetic, protein kinase inhibition and cytotoxic properties were assessed in vitro to compare the therapeutic potential of both kinds of TiO_2_ nanoparticles. All biological activities depicted significant enhancement as a result of addition of N as doping agent to TiO_2_ nanoparticles. *Klebsiella pneumoniae* has been illuminated to be the most susceptible bacterial strain out of various Gram-positive and Gram-negative isolates of bacteria used in this study. Good fungicidal activity has been revealed against *Aspergillus flavus*. 38.2% of antidiabetic activity and 80% of cytotoxicity has been elucidated by N-doped TiO_2_ nanoparticles towards alpha-amylase enzyme and *Artemia salina* (brine shrimps), respectively. Moreover, notable protein kinase inhibition against *Streptomyces* and antioxidant effect including reducing power and % inhibition of DPPH has been demonstrated. This investigation unveils the more effective nature of N-doped TiO_2_ nanoparticles in comparison to undoped TiO_2_ nanoparticles indicated by various biological tests. Hence, N-doped TiO_2_ nanoparticles have more potential to be employed in biomedicine for the cure of numerous infections.

## 1. Introduction

Biomedical applications of metallic oxide nanoparticles are recently considered an interesting avenue of research regarding nano-biotechnology. Nanoparticles possess unique physio-chemical, optical and electro-magnetic properties that make them appropriate tools for various applications in the field of materials science and pharmacy [1,2]. Co-precipitation is the most adaptive method for preparation of metallic oxide nanoparticles because of its reproducibility and cheapness [3]. Titanium dioxide (TiO_2_) nanoparticles have been vastly synthesized by different chemical routes. Doping prevents aggregation between nanoparticles ultimately leading to their stability, longevity as well as intensified reactivity [4]. Hence, TiO_2_ nanoparticles have been doped with Ni, Cu, Fe, Mo, N and other metals using hydrolysis, precipitation, sol-gel and other methods for fabrication in the past [5,6,7,8,9,10,11]. Metal-doped TiO_2_ nanoparticles serve many environmental remediation purposes like removal of organic dyes and wastes/pollutants owing to their tremendous photocatalytic activity [12]. In context of their role in biomedicine, antibacterial activity of Mn-doped TiO_2_ and Nd-doped TiO_2_ nanoparticles has been documented [13,14]. TiO_2_ nanoparticles have been proven important skin protecting agents against UV radiation and used in sunscreens/cosmetics as reported by Viana et al. [15]. Cytotoxicity of TiO_2_ nanoparticles doped with Ag, Cu, Fe has been validated against human cancer cell lines and hence these nanoparticles have been successfully declared anti-cancerous materials [16,17,18].

Regarding bioremediation studies, the photocatalytic property of N-doped TiO_2_ nanoparticles for air and water purification was exclusively described [19,20,21]. Talking about biomedical properties, Zane et al. [22] reported the biocompatibility and antibacterial activity of N-doped TiO_2_ nanoparticles and its applications in dental resin formulations. Moreover, the assessment of orthodontic brackets coated with N-doped TiO_2_ nanoparticles against *Streptococcus mutans* was illuminated by Salehi et al. [23]. Atchudan et al. [24] explained the applications of N-doped TiO_2_ nanoparticles in cell imaging due to their good biocompatibility. Li et al. [25] studied the photokilling effect of N-doped TiO_2_ nanoparticles on cancer cells.

The purpose of the present study was to complement the vacuum in literature regarding exploration of comprehensive biological potential of N-doped and undoped TiO_2_ nanoparticles. According to our knowledge, this is the first report covering multifunctional role of bare TiO_2_ nanoparticles and N-doped TiO_2_ nanoparticles in biomedicine. Antibacterial, antifungal, antioxidant, antidiabetic, protein kinase inhibition and cytotoxicity assays have been carried out under in vitro conditions. Comparative assessment of undoped and N-doped TiO_2_ nanoparticles has been performed to exploit the effectivity of these nanoparticles to be used as drug-loading agents or carriers in nanomedicine for the cure of countless fatal diseases disrupting human health.

## 2. Results and Discussion

### 2.1. X-Ray Diffraction (XRD)

**Figure 1:**
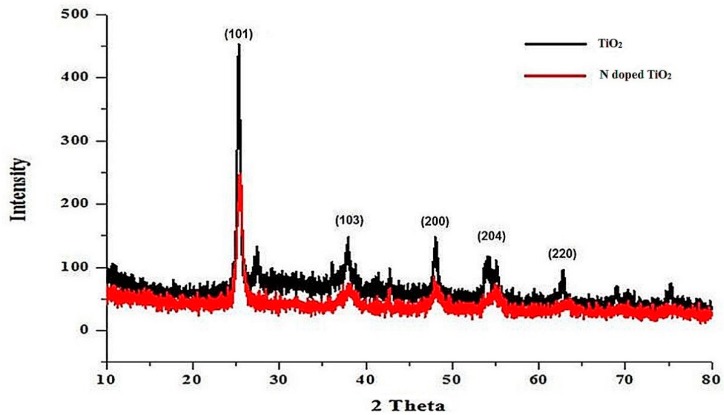
XRD pattern of TiO_2_ nanoparticles and N-doped TiO_2_ nanoparticles.

XRD data confirmed the nanocrystalline structure of TiO_2_ nanoparticles and N-doped TiO_2_ nanoparticles as evident from the sharpness of peaks obtained in Figure 1. The structure of TiO_2_ nanoparticles is tetragonal.

Later on, the size of nanoparticles was measured from the following Scherrer’s formula:

D = kλ/β cos θB(1)

Where λ indicates the wavelength of X-rays, β is the full width at half maximum of peaks in radians, and θB is the Bragg’s angle. The size of TiO_2_ nanoparticles and N-doped TiO_2_ nanoparticles was found to be 25 nm and 17.5 nm, respectively.

### 2.2. Scanning Electron Microscopy (SEM) and Energy Dispersive X-Ray (EDX)

**Figure 2:**
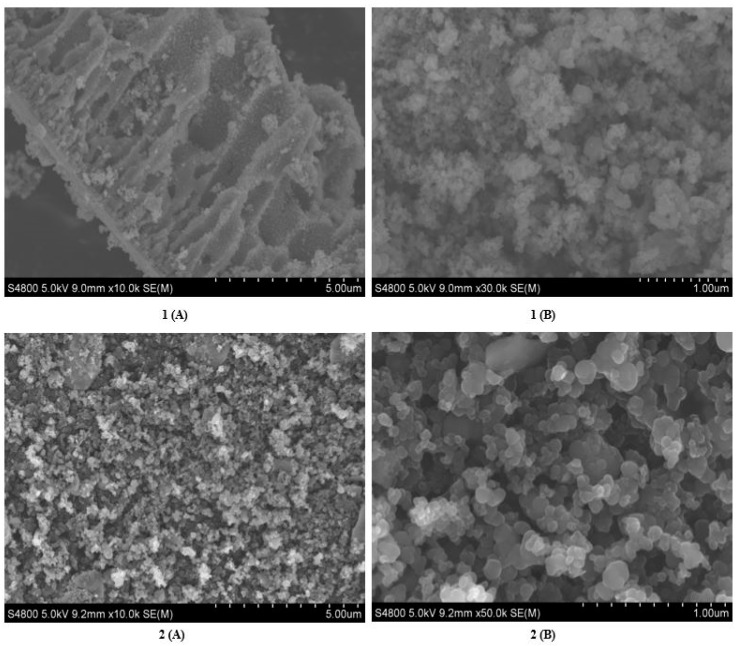
SEM **1**(**A**) low resolution image and **1**(**B**) high resolution image of TiO_2_ nanoparticles, and SEM **2**(**A**) low resolution image, and **2**(**B**) high resolution image of N-doped TiO_2_ nanoparticles.

The SEM micrographs in Figure 2 reveal spherical and coarse shape of TiO_2_ nanoparticles and N-doped TiO_2_ nanoparticles. However, morphology of TiO_2_ nanoparticles indicate more aggregation while crystals of N-doped TiO_2_ nanoparticles are aggregated less, showing better clarity of shape.

**Figure 3:**
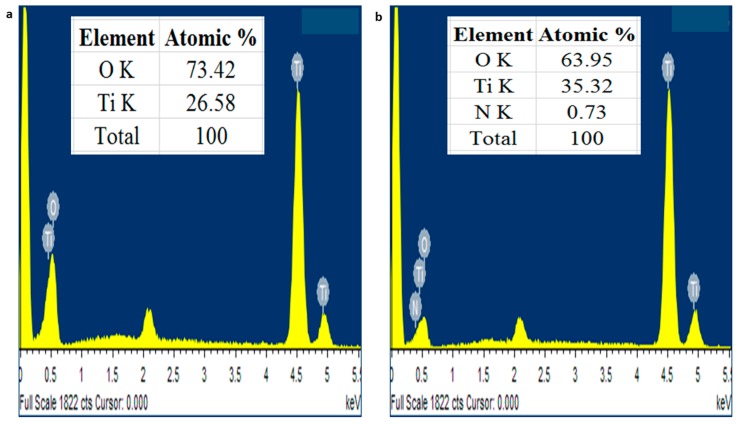
EDX pattern of (**A**) TiO_2_ nanoparticles and (**B**) N-doped TiO_2_ nanoparticles.

The EDX data in Figure 3 demonstrates that TiO_2_ nanoparticles were composed of only titanium and oxygen while N-doped TiO_2_ nanoparticles also had nitrogen found on the surface of TiO_2_. No other impurity was detected and these nanoparticles were found stoichiometric. Our results are concordant with the previous finding [26]. The SEM and EDX results were coinciding with XRD results as they also declared phase purity of TiO_2_ nanoparticles and N-doped TiO_2_ nanoparticles.

### 2.3. Transmission Electron Microscopy (TEM)

**Figure 4:**
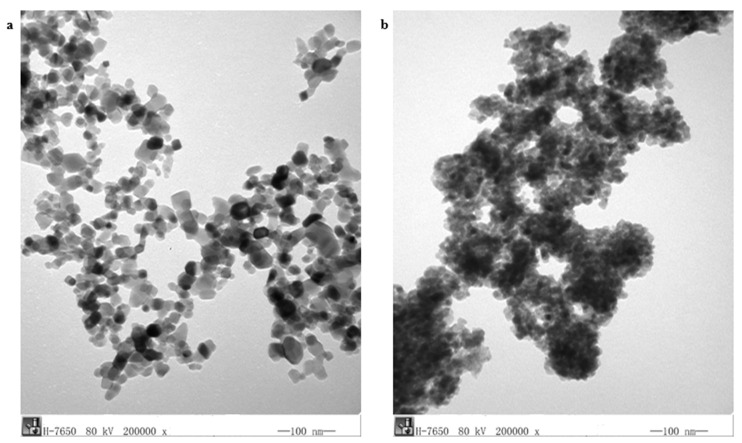
TEM image of (**a**) TiO_2_ nanoparticles and (**b**) N-doped TiO_2_ nanoparticles.

Figure 4 reveals the TEM micrographs of undoped TiO_2_ nanoparticles and N-doped TiO_2_ nanoparticles. The N doping has been confirmed indicated by the presence of N on the surface of TiO_2_ nanoparticles. The size of TiO_2_ nanoparticles and N-doped TiO_2_ nanoparticles has been found to be 20–25 nm and 10–15 nm, respectively.

### 2.4. Antibacterial Activity

**Figure 5:**
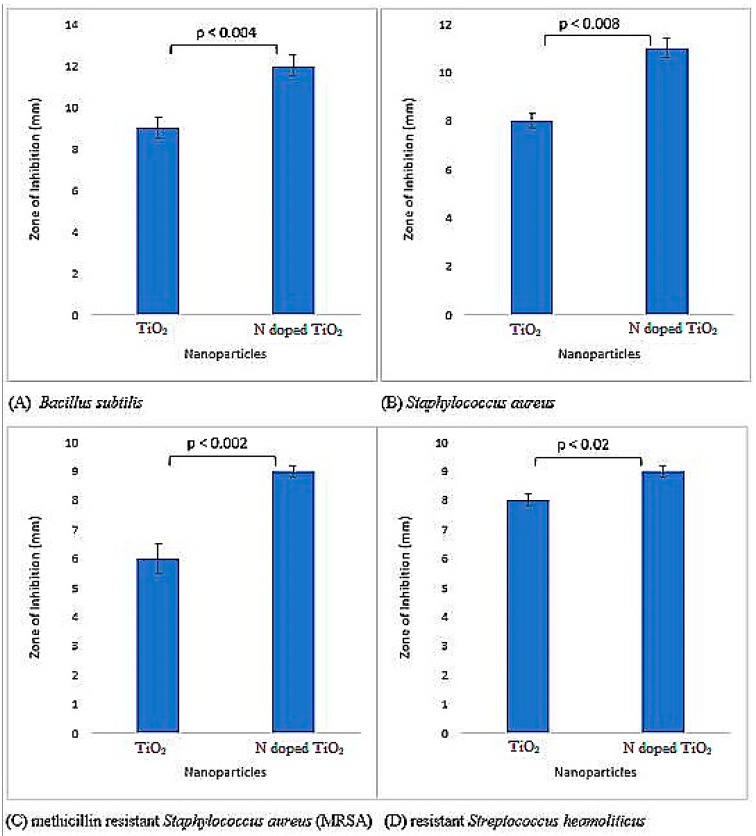
Antibacterial activity exhibited by TiO_2_ nanoparticles and N-doped TiO_2_ nanoparticles against Gram-positive bacterial strains; (A) *Bacillus subtillis*, (B) *Staphylococcus aureus*, (C) methicillin resistant *Staphylococcus aureus* (MRSA), (D) resistant *Streptococcus haemoliticus.* Error bars are shown as standard deviation on each bar. Bars are significantly different at confidence interval level of 95%.

**Figure 6:**
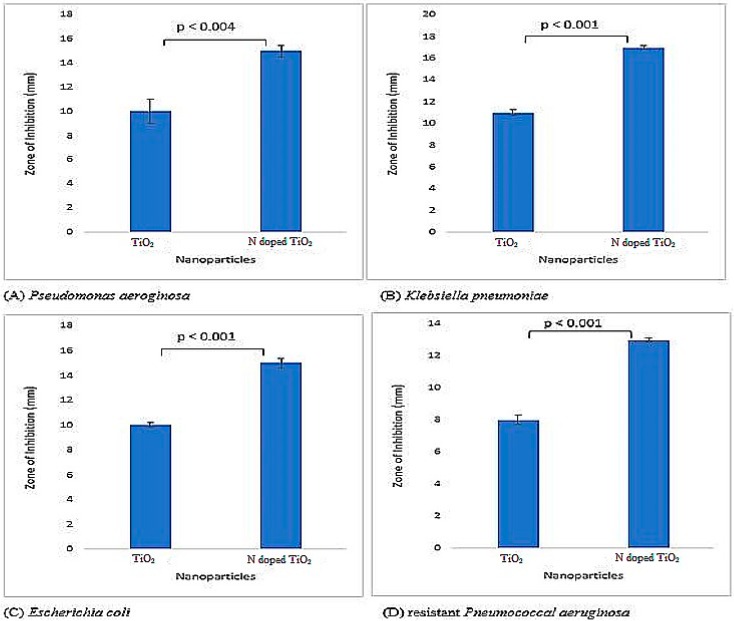
Antibacterial activity exhibited by TiO_2_ nanoparticles and N-doped TiO_2_ nanoparticles against Gram-negative bacterial strains; (**A**) *Pseudomonas aeroginosa*, (**B**) *Klebsiella pneumoniae*, (**C**) *Escherichia coli*, (**D**) resistant *Pneumococcal aeruginosa.* Error bars are shown as standard deviation on each bar. Bars are significantly different at confidence interval level of 95%.

**Figure 7:**
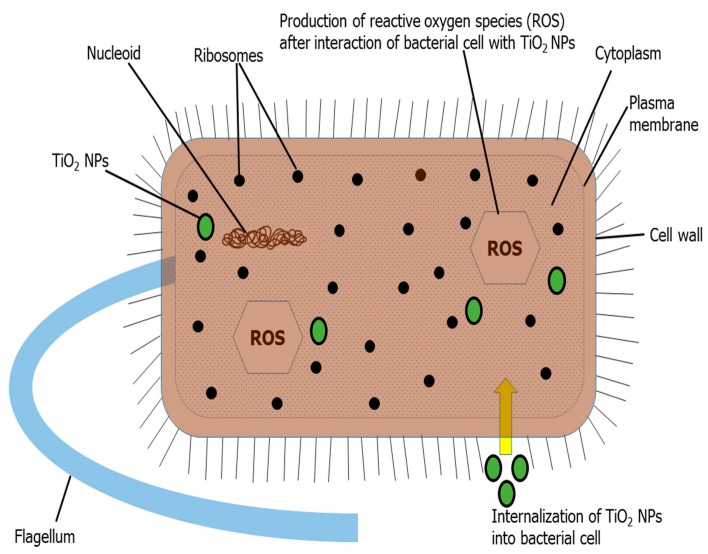
Diagrammatic illustration of mechanism of bactericidal activity of TiO_2_ nanoparticles.

Both bare TiO_2_ nanoparticles and N-doped TiO_2_ nanoparticles were tested for their antibacterial activity and found to be potent inhibitors of Gram-positive and Gram-negative bacteria as illustrated by Figure 5 and Figure 6. The literature has also documented TiO_2_ nanoparticles to be effective antibacterial agents [27,28,29,30,31]. There is difference in bacterial inhibition of different strains because of different functional groups present on their surface [4]. The positively charged nanoparticles and negatively charged bacterial surface leads to electrostatic interaction and hence, bactericidal activity by the formation of reactive oxygen species (ROS), that causes cell lethality after malfunctioning of bacterial synthetic machinery [31,32,33,34]. The mechanism for bactericidal activity of TiO_2_ nanoparticles has been shown in Figure 7.

The antibacterial activity against Gram-negative bacteria (*Pseudomonas aeruginosa*, *Klebsiella pneumoniae*, *Escherichia coli* and resistant *Pneumococcal aeruginosa*) was more pronounced as compared to Gram-positive bacteria (*Bacillus subtilis*, *Staphylococcus aureus*, methicillin resistant *Staphylococcus aureus* (MRSA) and resistant *Streptococcus haemoliticus*). The difference in cell wall composition and thickness of Gram-positive and Gram-negative bacteria might account for the variation in biological potential [4,35]. Furthermore, in the current scenario, it is believed that TiO_2_ nanoparticles more effectively permeate Gram-negative bacterial cell wall because of its lesser thickness via chemical bonding, and after translocation into the cytoplasm and nucleus, result in oxidative damage by excessive ROS production and eventual mortality. Therefore, Gram-negative bacteria are more vulnerable to TiO_2_ nanoparticles than their Gram-positive complement. Recently, Ripolles-Avila et al. [36] performed antibacterial studies of TiO_2_ nanoparticles and observed no significant difference in their behavior towards Gram-positive and Gram-negative bacteria.

The impact of doping agent was also prominent since the N-doped TiO_2_ nanoparticles showed more distinct bactericidal effect against all bacterial strains in comparison to undoped TiO_2_ nanoparticles. The highest antibacterial activity was elucidated by N-doped TiO_2_ nanoparticles against *Klebsiella pneumoniae* (zone of inhibition 17 mm) in this investigation. It is believed that doping causes reduction in nanoparticles’ size, their higher surface area, and ultimately more significant antibacterial activity [34,37]. Moreover, it has been proposed that enhanced antibacterial activity of N-doped TiO_2_ nanoparticles is due to the synergistic effect of N and TiO_2_ [4,35].

### 2.5. Antifungal Activity

**Figure 8:**
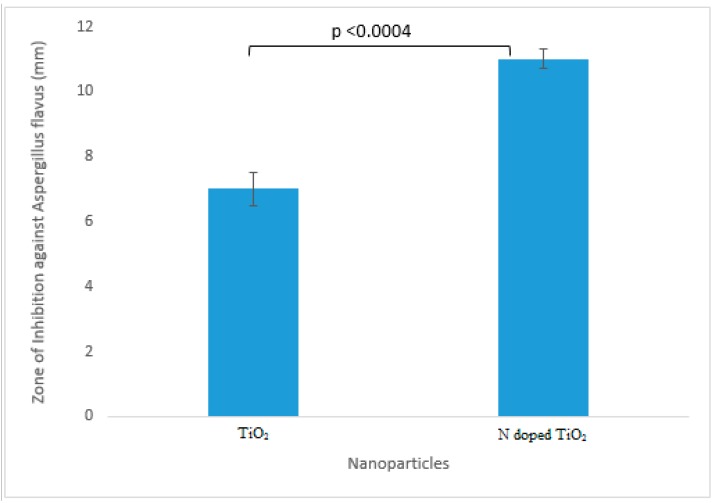
Antifungal activity exhibited by TiO_2_ nanoparticles and N-doped TiO_2_ nanoparticles against *Aspergillus flavus.* Error bars are shown as standard deviation on each bar. Bars are significantly different at confidence interval level of 95%.

**Figure 9:**
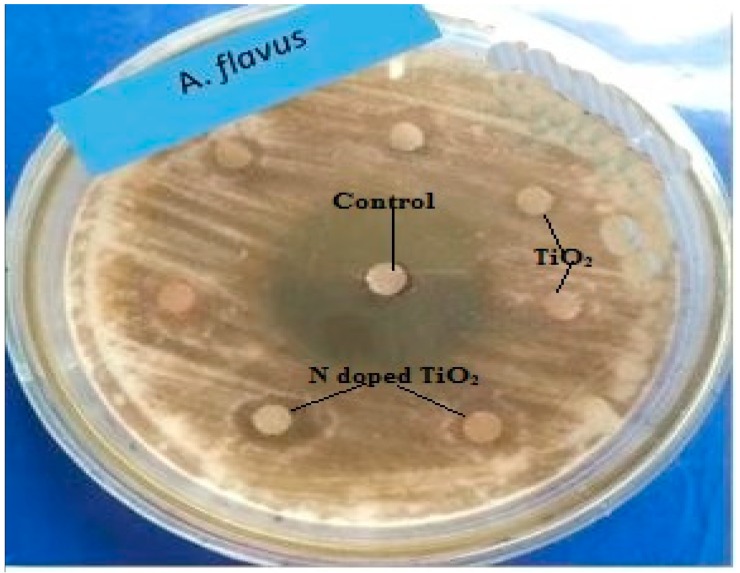
Antifungal activity: Zone of inhibition obtained against fungal strain, *Aspergillus flavus* by control, TiO_2_ nanoparticles and N-doped TiO_2_ nanoparticles.

The TiO_2_ nanoparticles tested for their antifungal potential against *Fusarium solani*, *Aspergillus flavus*, *Aspergillus fumigatus* and *Aspergillus niger* were found mildly effective against only *Aspergillus flavus* (zone of inhibition 7 mm and 11 mm for TiO_2_ nanoparticles and N-doped TiO_2_ nanoparticles, respectively) as evident from Figure 8 and Figure 9. Our study supports the previous report showing antifungal nature of TiO_2_ nanoparticles against *Candida albicans* [38]. The mechanism of fungicidal activity is not explicitly understood. However, it is thought to involve generation of ROS and oxidative deterioration causing cellular lethality [32,38].

### 2.6. Antioxidant Activity

**Figure 10:**
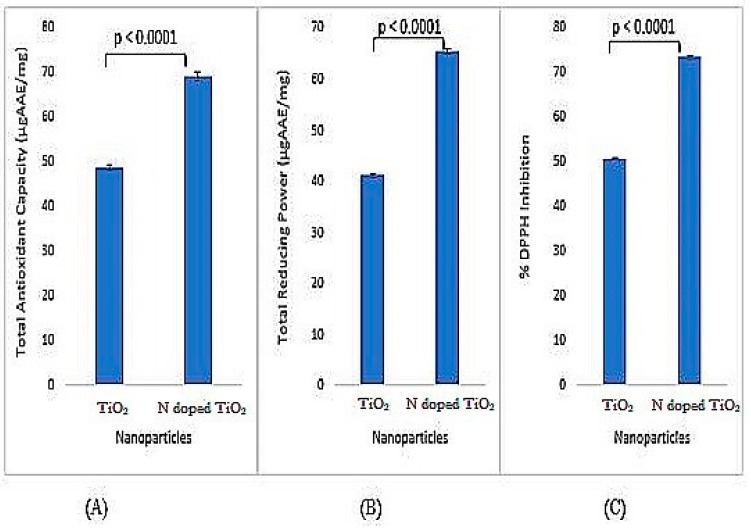
Antioxidant activity (**A**) total antioxidant capacity, (**B**) total reducing power, and (**C**) % DPPH inhibition exhibited by TiO_2_ nanoparticles and N-doped TiO_2_ nanoparticles. Error bars are shown as standard deviation on each bar. Bars are significantly different at confidence interval level of 95%.

All kinds of antioxidant activities, i.e., total antioxidant capacity (TAC), total reducing power (TRP) and % DPPH inhibition have been observed by both undoped TiO_2_ nanoparticles and N-doped TiO_2_ nanoparticles. Prominent antioxidation potential has been documented by the previous studies [27,29,30,39]. However, the activities shown by N-doped TiO_2_ nanoparticles are significantly higher than undoped TiO_2_ nanoparticles as depicted by Figure 10. A significant elevation in all activities in N-doped TiO_2_ nanoparticles is believed to be due to the addition of doping agent which reduces size of TiO_2_ nanoparticles and enhances their reactivity [4,35].

### 2.7. Antidiabetic Activity

**Figure 11:**
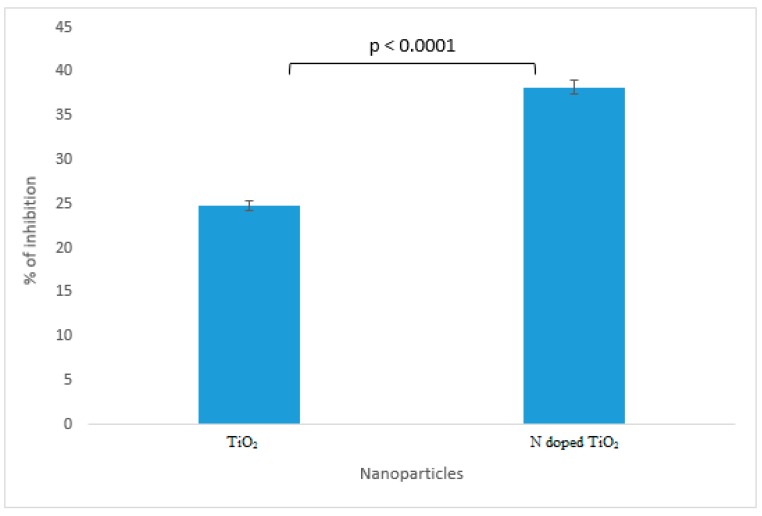
Antidiabetic activity exhibited by TiO_2_ nanoparticles and N-doped TiO_2_ nanoparticles against alpha amylase. Error bars are shown as standard deviation on each bar. Bars are significantly different at confidence interval level of 95%.

The results of α-amylase inhibition as shown in Figure 11 demonstrate undoped TiO_2_ nanoparticles and N-doped TiO_2_ nanoparticles to be the potent candidates against diabetes mellitus. Previously, *Stevia rebaudiana* loaded TiO_2_ nanoparticles have been proved potential antidiabetic agents via in vivo studies performed in rats [40]. N-doped TiO_2_ nanoparticles possess more significant α-amylase inhibition (38.2%) as compared to bare TiO_2_ nanoparticles (24.8%). The enlargement of surface area by size reduction of TiO_2_ nanoparticles results in increase of chemical bonding of surface atoms or molecules which paves the way for enhancement of antidiabetic activity in N-doped TiO_2_ nanoparticles [4,35].

### 2.8. Protein Kinase Inhibition Activity

**Figure 12:**
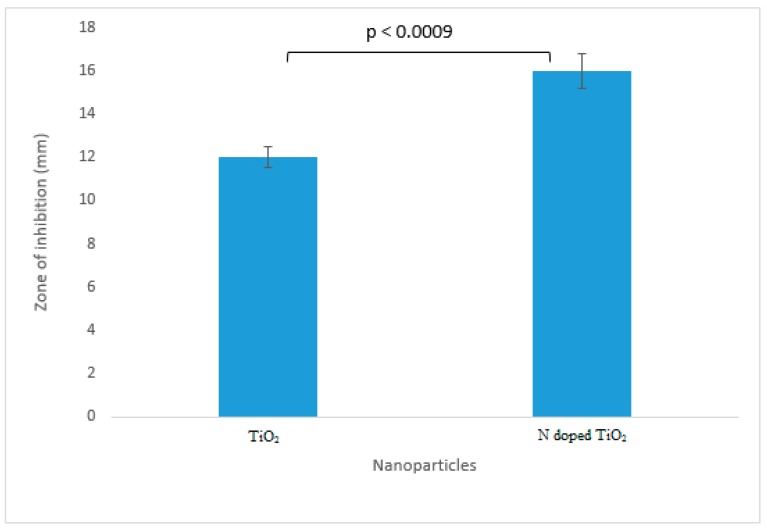
Protein kinase inhibition exhibited by TiO_2_ nanoparticles and N-doped TiO_2_ nanoparticles. Error bars are shown as standard deviation on each bar. Bars are significantly different at confidence interval level of 95%.

**Figure 13:**
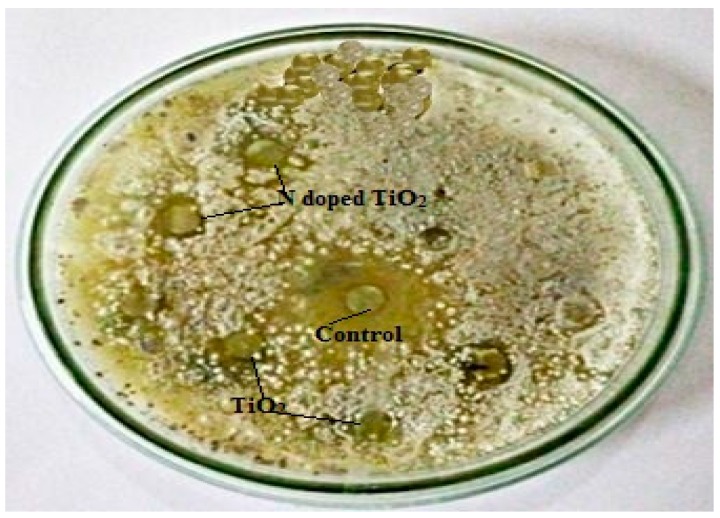
Protein kinase inhibitory activity: Zone of inhibition obtained against bacterial strain, *Streptomyces* by control, TiO_2_ nanoparticles and N-doped TiO_2_ nanoparticles.

The premier protein kinase inhibition was achieved by undoped TiO_2_ nanoparticles (inhibition zone 12 mm) and N-doped TiO_2_ nanoparticles (inhibition zone 16 mm) as deciphered by Figure 12 and Figure 13. It has been explicitly found that the highest protein kinase inhibition activity was demonstrated by N-doped TiO_2_ nanoparticles in comparison to their bare counterpart which might be due to the enhanced surface area of TiO_2_ nanoparticles after doping with N [4]. Protein kinase inhibition is the preliminary anticancer assay and previous reports have documented very good anticancer activity of TiO_2_ nanoparticles [41,42,43,44].

### 2.9. Cytotoxic Activity

**Figure 14:**
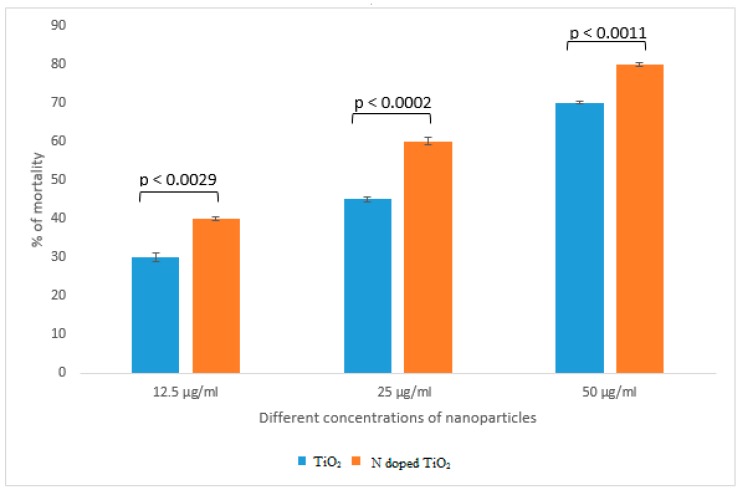
Cytotoxic activity exhibited by TiO_2_ nanoparticles and N-doped TiO_2_ nanoparticles. Error bars are shown as standard deviation on each bar. Bars are significantly different at confidence interval level of 95%.

Brine shrimp lethality assay was an appropriate preliminary test for screening of cytotoxicity of undoped TiO_2_ nanoparticles and N-doped TiO_2_ nanoparticles. Figure 14 shows that lesser cytotoxicity was obtained by bare TiO_2_ nanoparticles (70% of mortality at 50 µg/mL) as compared to N-doped TiO_2_ nanoparticles (80% of mortality at 50 µg/mL). Previous literature supports our findings by suggesting that TiO_2_ nanoparticles are cytotoxic to various human cell lines and doping further improves the cytotoxic status of these nanoparticles [16]. He et al. [45] elucidated concentration-dependent cytotoxic activity of TiO_2_ nanoparticles in human prostate cancer cell lines. Chellappa et al. [46], Ahamed et al. [15], Rahmani Kukia et al. [47] and Koca and Duman, [48] also reported the in vitro and in vivo cytotoxicity of TiO_2_ nanoparticles. The mechanism behind cytotoxicity might involve internalization of TiO_2_ nanoparticles into cytoplasm resulting in generation of ROS by oxidative stress and alteration in cellular redox state. It eventually causes mitochondrial damage and cell death by apoptosis [49,50,51].

The overall results of TiO_2_ nanoparticles and N-doped TiO_2_ nanoparticles with respect to their characterization techniques and therapeutic potential have been listed in Table 1.

## 3. Material and Methods

### 3.1. Chemical Fabrication of TiO_2_ Nanoparticles and N-Doped TiO_2_ Nanoparticles

In brief, TiO_2_ nanoparticles were synthesized by a facile chemical method of co-precipitation. The reagents used were purchased from Sigma-Aldrich (St. Louis, MO, USA) and included titanium isopropoxide (97%), isopropyl alcohol (≥98%), nitric acid (70%) and ethanol (96%). The procedure of Shajudheen et al. [52] was followed after some modifications. Synthesis of undoped TiO_2_ nanoparticles involved addition of 100 mL isopropyl alcohol to 15 mL titanium isopropoxide under continuous stirring for 30 min. 10 mL deionized water for hydrolysis was later added dropwise until white precipitates were obtained that were filtered and washed with deionized water and ethanol thrice. The precipitates were dried at 100 °C and grinded to a fine powder. Finally, calcination was done at 800 °C for 4 h.

In case of N-doped TiO_2_ nanoparticles synthesis, 15 mL nitric acid as a nitrogen source was added to titanium isopropoxide mixture and it was vigorously stirred until dissolved. Later on, deionized water was added followed by the procedure similar to undoped synthesis of TiO_2_ nanoparticles.

### 3.2. Characterization of TiO_2_ Nanoparticles and N-doped TiO_2_ Nanoparticles

#### 3.2.1. X-Ray Diffraction (XRD)

Powder x-ray diffraction (XRD) was performed for phase identification of nanoparticles. Bruker, D8 Advanced instrument (Bruker, Billerica, MA, USA) was used in 2θ range of 10–80° at 1.2/min scan rate. The radiation source used was Cu Kα (λ = 1.54056 Å) at 40 mA current and 40 kV voltage. Scherer’s equation was used to calculate the theoretical size of nanoparticles.

#### 3.2.2. Scanning Electron Microscopy (SEM) and Energy Dispersive X-Ray (EDX)

The shape and elemental composition of nanoparticles was revealed using scanning electron microscopy (SEM) and energy dispersive x-ray (EDX) spectroscopy. HITACHI S-4800 (HITACHI, Ibraraki, Japan) was used to determine SEM and EDX images.

#### 3.2.3. Transmission Electron Microscopy (TEM)

The transmission electron microscopy (TEM) was executed with HITACHI H-7650 (HITACHI, Japan) in order to find out an exact size of nanoparticles.

### 3.3. Biological Potential of TiO_2_ Nanoparticles and N-Doped TiO_2_ Nanoparticles

#### 3.3.1. Antibacterial Activity

The fabricated nanoparticles were subjected to screening for their antibacterial activity against various gram-positive and gram-negative bacterial strains. Agar disc diffusion method was employed for this purpose following the procedure of Haq et al. [53] after slight modifications. The Gram-positive bacterial isolates used were *Bacillus subtilis*, *Staphylococcus aureus*, methicillin resistant *Staphylococcus aureus* (MRSA) and resistant *Streptococcus haemoliticus*. Whereas, *Pseudomonas aeruginosa*, *Klebsiella pneumoniae*, *Escherichia coli* and resistant *Pneumococcal aeruginosa* were the Gram-negative isolates of bacteria used for finding antibacterial potential. All of strains were allowed to grow on 2% nutrient agar (NA) at 25 °C and stored in refrigerator. Then, 100 µL of tryptic soy broth (TSB) was spread on agar medium in which bacterial colonies were grown. Stock solution of 1 mg/mL of nanoparticles was made in dimethyl sulfoxide (DMSO), from which 50 µg/mL of final concentration was made by taking out 50 µL of solution and loading on discs found on petri plates. The incubation of plates was done at 37 °C for 24 h. DMSO were used as negative control, whereas Roxithromycin and Cefixime were taken as positive controls. Vernier caliper was used to measure inhibition zones.

#### 3.3.2. Antifungal Activity

Agar disc diffusion method was used by following the procedure of Akhtar et al. [54] after slight modifications to evaluate antifungal activity of nanoparticles. The fungal strains used were *Fusarium solani*, *Aspergillus flavus*, *Aspergillus fumigatus* and *Aspergillus niger*. All strains were preserved in refrigerator at 4 °C on sabouraud dextrose agar (SDA). After culturing of fungal strains on SDA, 50 µg/mL of nanoparticles solution was loaded on discs found on petri plates containing media. The prepared petri dishes were incubated at 28 °C for 72 h. DMSO and Clotrimazole were used as negative control and positive control, respectively. The zones of inhibition were measured with Vernier caliper.

### 3.4. Antioxidant Activity

#### 3.4.1. Total Antioxidant Capacity (TAC)

TAC of nanoparticles was elucidated by some modifications in the method of Khan et al. [55]. 4mg/mL stock solution of nanoparticles was made from which 100 µL was taken out and mixed with 900 µL of reagent solutions (4 mM ammonium molybdate, 28 mM sodium phosphate and 0.6 M sulfuric acid). After incubation at 95 °C for 90 min, absorbance was taken at 695 nm using microplate reader. DMSO and Ascorbic acid were taken as negative control and positive control, respectively. The results were expressed as µg AA/mg.

#### 3.4.2. Total Reducing Power (TRP)

TRP of nanoparticles was evaluated by some modifications in the method of Khan et al. [55]. From 4mg/mL stock nanoparticles solution, 100 µL was mixed with reagents (200 µL of phosphate buffer and 250 µL of 1% potassium ferricyanide). After incubation at 50 °C for 20 min, 200 µL of 10% trichloroacetic acid was added. Centrifugation (LB-5, Heraeus, Hanau, Germany) was carried out at 3000 rpm for 10 min. Later on, 150 µL of supernatant solution was taken and mixed with 50 µL of 0.1% ferric chloride solution. Finally, absorbance was measured at 630 nm using microplate reader. DMSO and Ascorbic acid were used as negative control and positive control, respectively. The results were expressed as µg AA/mg.

### 3.5. % DPPH Inhibition

Free radical scavenging activity of nanoparticles was determined using 2,2-diphenyl-1-picryl hydrazyl (DPPH) reagent by some modifications in the method of Khan et al. [55]. 10 µL of nanoparticles solution was taken from 4mg/mL stock solution and mixed with 190 µL of DPPH reagent. After incubation for 1 h in dark, absorbance was measured at 515 nm with microplate reader. DMSO and Ascorbic acid were used as negative control and positive control, respectively.

% free radical scavenging or % inhibition of nanoparticles with DPPH was calculated using the following formula:

% DPPH Inhibition = (1 − Abs/Abc) × 100(2)

where Abs is the absorbance of nanoparticles with DPPH reagent, and Abc is the absorbance of negative control. Table curve software 2D Ver. 4 (SPSS Inc., Chicago, IL., USA) was used to calculate IC50.

### 3.6. Antidiabetic Activity

Antidiabetic activity or α-amylase inhibition was determined by a method described by [56] after slight modifications. It involved addition of 15 µL of phosphate buffer, 25 µL of α-amylase, 10 µL of nanoparticle solution and 40 µL of starch to the well of microtiter plate. The plate was incubated at 50 °C for 30 min. 20 µL of 1 M HCl and 90 µL of iodine solution were added thereafter. DMSO and Acarbose were taken as negative control and positive control, respectively. DMSO, starch and buffer were taken as blank. Microplate reader measured the absorbance at 540 nm.

% inhibition of α-amylase was calculated by the following formula:

% enzyme inhibition = (ODx − ODy/ODz − ODy) × 100(3)

where ODx, ODy and ODz symbolize the absorbance of nanoparticles, negative control and blank, respectively.

### 3.7. Protein Kinase Inhibition Activity

The method established by Yao et al. [57] after slight modifications was utilized to determine preliminary antitumor activity of nanoparticles by means of protein kinase inhibition assay. It was based on the principle of inhibition in hyphae formation in *Streptomyces* whose mycelia fragments were allowed to disperse on the surface of ISP4 plates of agar media. 5 µL of nanoparticles solution was applied to these plates and placed for incubation for 72 h. The clear or bald zones appeared after incubation were measured using Vernier caliper. The bald zone more than 9 mm is considered significant and indication of restriction in bacterial hyphae formulation. DMSO and Surfactin were taken as negative control and positive control, respectively.

### 3.8. Cytotoxic Activity

Brine shrimp lethality test was performed to reveal the cytotoxicity of nanoparticles by a method of Apu et al. [58] after minor modifications. The *Artemia salina* (brine shrimp) eggs were hatched in a tank containing artificial sea water having incubation temperature of 30 °C. The tank was comprised of two unequal portions; the larger portion contained eggs and was sealed with aluminium foil, while the smaller portion had light source due to which the newly hatched larvae of shrimps gathered there within 1–2 days. The mature nauplii were shifted in a small beaker using Pasteur pipette. Different dilutions from stock solutions (4 mg/mL) of nanoparticles at concentrations of 50 µg/mL, 25 µg/mL and 12.5 µg/mL were tested for assessment of lethality. Each well of 96-well plate were filled with 10 nauplii and 150 µL of sea water in which the dilutions were poured that raised the final volume to 300 µL. The resulting solutions were analyzed for quantification of % of dead nauplii after 2 h of incubation period. DMSO and Doxorubicin were used as negative control and positive control, respectively. Table curve 2 D Ver. 4 software was used to calculate LC50 of nanoparticles.

### 3.9. Statistical Analysis

All tests were performed in triplicate and data were presented as mean ± standard deviation (SD) from at least three replicates.

## 4. Conclusions

Although there is abundant data available on the investigation of biological uses of TiO_2_ nanoparticles, rare content is found regarding study of comparative effects of doping agent/surfactant on the biological nature of these nanoparticles. Therefore, this study was intended to estimate the comprehensive therapeutic potential of synthesized undoped and N-doped TiO_2_ nanoparticles via different biological activities performed in vitro. All bioassays have proved the efficiency of N-doped TiO_2_ nanoparticles and good/moderate biological activities have been achieved. In short, these nanoparticles are highlighted as tools for use in the treatment of various human diseases. However, further extensive attempts on cytotoxic and antitumor inquiry of these nanoparticles on the normal human cells are strongly recommended.

## Figures and Tables

**Table 1:** Summary of results of TiO_2_ nanoparticles and N-doped TiO_2_ nanoparticles with respect to their characterization techniques and therapeutic potential.

Methods of Characterization and Biological Potential	TiO_2_ Nanoparticles	N-doped TiO_2_ Nanoparticles
**Characterization**		
XRD	Size: 25 nm	Size: 17.5 nm
SEM	Morphology: Spherical but lesser clarity of shape due to more aggregation	Morphology: Spherical and more clarity of shape due to lesser aggregation
EDX	O: 73.42%	O: 63.95%
	Ti: 26.58%	Ti: 35.32%
	-	N: 0.73%
TEM	Size: 20–25 nm	Size: 10–15 nm
**Biological Potential**		
Antibacterial activity against Gram-positive bacteria:	Zone of Inhibition Gram-positive bacteria:	Zone of Inhibition Gram-positive bacteria:
*Bacillus subtilis*	9 mm	12 mm
*Staphylococcus aureus* *methicillin resistant*	8 mm	11 mm
*Staphylococcus aureus resistant*	6 mm	9 mm
*Streptococcus haemoliticus*	8 mm	9 mm
**Gram-negative bacteria:**	**Gram-negative bacteria:**	**Gram-negative bacteria:**
*Pseudomonas aeruginosa*	10 mm	15 mm
*Klebsiella pneumoniae*	11 mm	17 mm
*Escherichia coli resistant*	10 mm	15 mm
*Pneumococcal aeruginosa*	8 mm	13 mm
Antifungal activity against *Aspergillus flavus*	Zone of Inhibition: 7 mm	Zone of Inhibition: 11 mm
**Antioxidant activity**		
TAC	48.9 µgAAE/mg	69.1 µgAAE/mg
TRP	41.2 µgAAE/mg	65.5 µgAAE/mg
% DPPH Inhibition	50.8%	73.6%
Antidiabetic activity against alpha amylase enzyme	% of Inhibition: 24.8%	% of Inhibition: 38.2%
Protein kinase inhibitory activity	Zone of Inhibition: 12 mm	Zone of Inhibition: 16 mm
**Cytotoxic activity at**	**% of mortality**	**% of mortality**
12.5 µg/mL	30%	40%
25 µg/mL	45%	60%
50 µg/mL	70%	80%
